# Interments in the City and Liberties of Philadelphia, from the 10th of July, to the 17th

**Published:** 1841-07-24

**Authors:** 


### HEALTH OF THE CITY

INTERMENTS *in the City and Liberties of Philadelphia, from the* 10*th of July, to the* 17*th.*

| Diseases. | Adults. | Children. |
|---|---|---|
| Anemia, | 1 | 0 |
| Cancer of the Stomach, | 1 | 0 |
| Casualty, | 0 | 1 |
| Croup, | 0 | 2 |
| Congestion of Lungs, | 0 | 1 |
| —— Brain, | 1 | 0 |
| Consumption of the lungs, | 10 | 2 |
| Convulsions, | 1 | 9 |
| Canerum Oris, | 0 | 1 |
| Diarrhœa, | 1 | 4 |
| Dropsy, | 2 | 1 |
| —— Head, | 0 | 2 |
| —— Heart, | 1 | 0 |
| Diseae of Lungs, | 1 | 0 |
| —— Bowels, | 1 | 1 |
| Drowned, | 1 | 0 |
| Dysentery, | 1 | 7 |
| Debility, | 0 | 2 |
| Drinking cold water, | 1 | 0 |
| Epilepsy, | 1 | 0 |
| Fever, | 0 | 1 |
| —— Bilious, | 1 | 0 |
| —— Typhus, | 1 | 0 |
| Hernia, | 1 | 0 |
| Inflammation of the Brain, | 1 | 5 |
| —— Bronchi, | 1 | 2 |
| —— Lungs, | 2 | 1 |
| —— Stomach, | 0 | 1 |
| —— Stomach and Bowels, | 0 | 1 |
| —— Bowels, | 1 | 4 |
| —— Liver, | 1 | 0 |
| —— Larynx, | 0 | 1 |
| —— Peritoneum, | 0 | 1 |
| | — | — |
| *Carried forward,* | 33 | 50 |

| Diseases | Adults. | Children. |
|---|---|---|
| *Brought forward,* | 33 | 50 |
| Intemperance, | 1 | 0 |
| Intestinal Irritation, | 0 | 1 |
| Jaundice, | 0 | 1 |
| Marasmus, | 0 | 4 |
| Measles, | 0 | 2 |
| Neglect, | 0 | 1 |
| Old age, | 2 | 0 |
| Pleurisy, | 2 | 0 |
| Scrofula, | 0 | 1 |
| Small pox, | 1 | 1 |
| Still-born, | 0 | 3 |
| Suicide, | 2 | 0 |
| Summer Complaint, | 0 | 34 |
| Unknown, | 1 | 1 |
| | — | — |
| Total, 141— | 42 | 99 |

Of the above, there were under 1 year, 52

| | |
|---|---|
| From 1 to 2 | 22 |
| 2 to 5 | 16 |
| 5 to 10 | 5 |
| 10 to 15 | 2 |
| 15 to 20 | 2 |
| 20 to 30 | 13 |
| 30 to 40 | 6 |
| 40 to 50 | 5 |
| 50 to 60 | 7 |
| 60 to 70 | 4 |
| 70 to 80 | 5 |
| 80 to 90 | 2 |
| 90 to 100 | 0 |
| Total, | 148 |

Of the above there were 6 from the almshouse, 22 people of colour, and 1 from the country, which are included in the total amount.

